# Intrinsically Disordered Proteins in Chronic Diseases

**DOI:** 10.3390/biom9040147

**Published:** 2019-04-11

**Authors:** Prakash Kulkarni, Vladimir N. Uversky

**Affiliations:** 1 Department of Medical Oncology and Therapeutics Research, City of Hope National Medical Center, Duarte, CA 91010, USA; 2 Department of Molecular Medicine, Morsani College of Medicine, University of South Florida, Tampa, FL 33612, USA; 3 Laboratory of New Methods in Biology, Institute for Biological Instrumentation, Russian Academy of Sciences, 142290 Pushchino, Moscow Region, Russia

It is now increasingly evident that a large fraction of the human proteome comprises proteins that, under physiological conditions, lack fixed, ordered 3D structures as a whole or have segments that are not likely to form a defined 3D structure [1,2,3,4,5,6,7]. These proteins and regions are referred to as intrinsically disordered proteins (IDPs) and intrinsically disordered protein regions (IDPRs), respectively. Despite their lack of a stable structure, IDPs/IDPRs are involved in a multitude of crucial biological functions related to regulation, recognition, signaling, and control, where binding to multiple partners and high-specificity/low-affinity interactions plays a crucial role [8,9,10,11,12,13,14]. Furthermore, intrinsic disorder is a unique structural feature that enables IDPs/IDPRs to participate in both one-to-many and many-to-one signaling [9,10]. Since they serve as general regulators of various cellular processes, IDPs/IDPRs themselves are tightly controlled [15,16]. However, when misexpressed, misprocessed, mismodified, or dysregulated, IDPs/IDPRs are prone to engage in promiscuous, often unwanted interactions and, thus, are associated with the development of various pathological states. In fact, the majority of human cancer-related proteins [8], as well as many proteins associated with neurodegeneration [17,18], diabetes [19], cardiovascular disease [20], amyloidosis [21], and genetic diseases [22], are either intrinsically disordered or contain long IDPRs. This broad involvement of misbehaving IDPs/IDPRs in human diseases is known as “disorder in disorders” (or D^2^) concept [23,24].

It is generally believed that IDPs/IDPRs stochastically sample all possible configurations driven by thermal fluctuations. While this may be true for many extended IDPs, which behave as “random” coils, it is likely that due to the variability in interaction energy between different amino acid sequences, some configurations may be strongly preferred while others are forbidden. Furthermore, unlike folded proteins that exhibit a high degree of structural order and undergo collective motions, albeit fairly constrained, IDPs/IDPRs also exhibit some structural and dynamical ordering, being much less constrained in their motions than folded proteins. Thus, the larger structural plasticity of IDPs emphasizes the importance of entropically driven motions. Because of their simplified spatial organization and globally reduced structural content, IDPs/IDPRs are characterized by the exceptional spatiotemporal heterogeneity, where different parts of a protein are ordered (or disordered) to a different degree, and this distribution is constantly changing over time [25,26]. Therefore, IDPs/IDPRs are not homogeneous, but have a very complex mosaic architecture reflecting their highly heterogeneous spatiotemporal structural organization that includes foldons (independently foldable units of a protein), inducible foldons (disordered regions that can fold at least in part due to their interaction with binding partners), non-foldons (non-foldable protein regions), semi-foldons (regions that are always in a semi-folded form), and unfoldons (ordered regions that have to undergo an order-to-disorder transition to become functional) [25,26]. Since these differently (dis)ordered structural elements might have well-defined and specific functions, an IDP/IDPR can be multifunctional, being involved in interaction with, regulation of, and be controlled by a multitude of structurally unrelated partners [27].

Although high-specificity/low-affinity interactions are considered a functional hallmark of IDPs/IDPRs, these proteins can also interact with ultrahigh (picomolar) affinity, but fully retain their structural disorder, long-range flexibility, and highly dynamic character [28]. Such structures, wherein bound IDPs/IDPRs retain structural plasticity, have been defined as fuzzy complexes [29,30]. While a large number of fuzzy complexes have been characterized, many of such interactions are between an IDP/IDPR and a structured protein. There are also examples of direct interactions between IDPs/IDPRs leading to the formation of a fuzzy complex without disorder-to-order transition [31,32,33]. With recent advances in experimental techniques and better integration with computational simulations, the picture that is emerging suggests that the modest structural ordering and large amplitude collective motions of IDPs/IDPRs enable them to mediate multiple interactions with different partners in the cell.

This Special Issue of *Biomolecules* is dedicated to elucidating how conformational dynamics affects interactions of IDP/IDPR with partner proteins, especially in those IDPs that are implicated in chronic diseases. Because of the unique properties they possess, and also because they are not amenable to classical tools and methods used to study ordered proteins, the IDPs/IDPRs have attracted attention across disciplines and scales. In fact, increasing curiosity in understanding how these fascinating molecules that lack unique structure but carry out the myriad functions resulted in an explosive expansion of the disorder-related literature, and scientists from various backgrounds as diverse as protein biochemists, classical physics, polymer physics, biophysics, theoretical physics, bioengineering, and computational and information science have begun to unravel many mysteries of IDPs/IDPRs. Technological advances, and the availability of more and more sophisticated computational tools, have further contributed to a deeper understanding of how IDPs are able carry out their functions. Therefore, it is not surprising that the various contributions to this Special Issue are from experts in biology, bioinformatics, biochemistry, biophysics, structural biology, and computational physics. 

The contributions by Bignon et al. ‘Modulation of measles virus NTAIL interactions through fuzziness and sequence features of disordered binding sites’ [34], and by Kumar and Thompson ‘Role of phosphorylation in the modulation of glucocorticoid receptor’s intrinsically disordered domain’ [35] provide an overview of the disorder-based functionality mostly from a biochemical perspective. Bignon et al. review recent findings on the different interaction mechanisms of the C-terminal domain of the intrinsically disordered nucleoprotein (N) of measles virus (MeV) N_TAIL_, with two of its known binding partners, namely, the C-terminal X domain of the phosphoprotein of MeV XD (a globular viral protein) and the heat-shock protein 70 (hsp70, a globular cellular protein). Kumar and Thompson, on the other hand, delve in the important role of phosphorylation in gene regulation by glucocorticoid receptor (GR) [34]. The GR N-terminal domain is highly disordered and undergoes disorder-to-order transition following site-specific phosphorylation. And this transition from disorder to order is critical for AF1′s efficient interaction with several coregulatory proteins and subsequent AF1-mediated GR activity [35]. 

Three articles comprise the structural biology section. Of course, protein NMR remains the work horse for gaining insight into IDP/IDPR (un)structure/function relationships. Levy et al., in their article ‘p53 phosphomimetics preserve transient secondary structure but reduce binding to Mdm2 and MdmX’ [36], examined the disordered p53 transactivation domain (p53TAD) using a combination of biophysical techniques including NMR. The p53TAD contains transient helical structures that are necessary for its binding to the negative regulators, mouse double minute 2 (Mdm2) and MdmX. The second paper in this section, ‘Conserved glycines control disorder and function in the Cold-Regulated Protein, COR15A’ by Sowemimo et al. [37], analyzes the plant cold-regulated protein COR15A from *Arabidopsis* that is important for freeze tolerance. During freezing-induced cellular dehydration, COR15A transitions from a disordered to mostly α-helical structure. The authors tested whether mutations that increase the helicity of COR15A also increase its protective function using a combination of NMR, circular dichroism, and fluorescence spectroscopy. The results of these experiments showed that the mutants with higher content of α-helical structure were characterized by an increased membrane stabilization potential during freezing [37]. Finally, the paper by Melková et al. ‘Structure and functions of microtubule associated proteins tau and MAP2c: similarities and differences’ [38] used a combination of NMR and cryo-electron microscopy to examine the propensities of MAPs, tau40 and MAP2, to form transient local structures and long-range contacts in the free state, and conformations adopted by these proteins in complexes with microtubules and filamentous actin, as well as in pathological aggregates. For both molecules, the authors identified transient structural motifs by conformational analysis of the experimental data and observed that many of the short sequence motifs that exhibit transient structural features are linked to functional properties, manifested by specific interactions. Therefore, this detailed structure–function analysis may help in explaining the observed differences between biological activities of tau40 and MAP2c [38].

Single-molecule fluorescence resonance energy transfer (or smFRET) is yet another powerful tool used to study IDPs/IDPRs. It is one of the few approaches that are sensitive to transient populations of substrates within molecular ensembles, and therefore, is ideally suited to discern conformational preferences of IDP/IDPR ensembles. The first paper in the section, ‘Spontaneous switching among conformational ensembles in intrinsically disordered proteins’, by Choi et al. [39] describes a growing number of proteins that appear intrinsically disordered by biochemical and bioinformatics characterization but switch between restricted regions of conformational space. Such switching between disparate corners of conformational space could bias ligand binding and regulate the volume of IDPs acting as structural or entropic elements. Therefore, mapping the accessible energy landscape and capturing dynamics across a wide range of timescales are essential for recognition of when an IDP/IDPR is acting as such a switch [39]. In the paper entitled ‘Extreme fuzziness: Direct interactions between two IDPs’, Wang and Wang [40] examined whether two IDPs can interact directly to form a fuzzy complex without disorder-to-order transition. Using a combination of smFRET, NMR, and molecular dynamics (MD) simulation, the authors demonstrate that direct interactions between the two pairs of IDPs, 4.1G-CTD/NuMA and H1/ProTα, do form fuzzy complexes while retaining high conformational dynamics of the isolated proteins, which they name as the extremely fuzzy complexes. Therefore, extreme fuzziness completes the full spectrum of protein–protein interaction modes, suggesting that a more generalized model beyond existing binding mechanisms is required [40]. 

Using a different biophysical approach, confocal fluorescence microscopy, Kaur et al. [41] in their paper entitled ‘Molecular crowding tunes material states of ribonucleoprotein condensates’, study how molecular crowding impacts ribonucleoprotein (RNP) liquid condensation using an archetypal disordered RNP, called fused in sarcoma (FUS), as an example. RNP condensation is largely governed by promiscuous attractive inter-chain interactions mediated by low-complexity domains (LCDs). The authors demonstrate that the liquid–liquid coexistence boundary of FUS is lowered by polymer crowders, consistent with an excluded volume model. With increasing bulk crowder concentration, the RNP partition increases and the diffusion rate decreases in the condensed phase. These results reveal that the impact of crowding is largely independent of LCD charge and sequence patterns. These results are consistent with a thermodynamic model of crowder-mediated depletion interaction, which suggests that inter-RNP attraction is enhanced by molecular crowding [41]. 

Characterizing the structure–function relationship of IDPs/IDPRs is no doubt an essential but daunting task as they can adapt transient structure. Molecular dynamics simulations (MDS) has emerged as a natural complement to various experimental approaches for atomic-level characterizations and mechanistic investigations of this intriguing class of proteins. This SI includes three interesting papers that have exploited this computational technique in conjunction with experimental data to gain new insight into the IDP/IDPR structure/function paradigm. In the first article in this section, ‘Electrostatics of Tau protein by molecular dynamics’, Castro et al. [42] employed MDS to study the structure of a microtubule associated protein Tau that promotes microtubule assembly and stability. To date, the 3D structure of Tau has not been fully solved, experimentally. This is the first MDS study of full-length Tau in conjunction with a region from the microtubule tubulin with which it interacts. The results bring a new insight into Tau and tubulin proteins, their characteristics and structure-function relationship, and highlights the fact that Tau is a disordered protein with discrete portions of well-defined secondary structure mostly at the microtubule binding region [42]. 

The second paper, ‘Structural and dynamical order of a disordered protein: Molecular insights into conformational switching of PAGE4 at the systems level’, describes another MDS study by Lin et al. [43]. Using prostate-associated gene 4 (PAGE4), an IDP implicated in prostate cancer (PCa), as an example, the authors describe the quantitative reproduction of experimental observations and reveal how structural and dynamic ordering are encoded in the sequence of PAGE4, and how these features can be modulated by different extents of phosphorylation by different kinases. This ordering is reflected in changing populations of certain secondary structural elements, as well as in the regularity of its collective motions, and correlate with the functional interactions of the different conformational ensembles of PAGE4 to give rise to repeated transitions between cellular phenotypes with important physiological consequences [43].

The third paper in this section on MDS and biophysical computation, ‘Recent advances in computational protocols addressing intrinsically disordered proteins’ by Bhattacharya and Lin [44], argues that to understand the conformational dynamics of IDPs/IDPRs and how their structural ensembles recognize multiple binding partners and small molecule inhibitors, knowledge-based and physics-based sampling techniques, guided by the experimental structural data, can be utilized for the comprehensive and focused in silico analyses. However, efficient sampling of the IDP/IDPR conformational ensemble requires traversing the numerous degrees of freedom in the IDP/IDPR energy landscape, as well as force-fields that accurately model the protein and solvent interactions. Therefore, these authors provide an overview of the current state of computational methods for studying IDP/IDPR structure and dynamics and discuss the major challenges in this field.

Finally, the paper ‘Functional segments on intrinsically disordered regions in disease-related proteins’ by Anbo et al. [45] describes a bioinformatics approach to study IDPs/IDPRs. IDPRs, which are often found in the ordered proteins, are known to play important roles in signaling pathways and transcriptional regulation. Therefore, the authors performed a bioinformatics analysis and found more than a thousand potential functional IDPR segments in disease-related proteins, which are found in cancers, congenital disorders, digestive system diseases, and reproductive system diseases. A detailed analysis of some of these regions showed that the functional segments are located on experimentally verified IDPRs. Since IDPs involved in disease pathology tend to have numerous protein–protein interactors, these data suggest that, by occupying hub positions in the protein–protein interaction networks, IDPs can have huge impacts on human diseases. This study highlights the utility of bioinformatics approaches in conjunction with experimental data to in casting new light on the IDPs/IDPRs [45].

In summary, we trust these articles on the IDPs/IDPRs will not only serve as excellent references, but will also stimulate a flurry of activity toward gaining a deeper insight into these fascinating molecules that constitute a large fraction of the proteomes across all three kingdoms of life and engage in myriad biological activities in ways that seem to challenge conventional wisdom. With new advances in experimental techniques, theoretical concepts, and computational capabilities that permit observations across spatiotemporal scales, it is likely that we may have a better understanding of the IDPs and be able to design strategies to target them for therapeutic purposes.
